# Effect of Bridging Manner on the Transport Behaviors of Dimethyldihydropyrene/Cyclophanediene Molecular Devices

**DOI:** 10.3390/molecules29122726

**Published:** 2024-06-07

**Authors:** Peng Cui, Zhouhao Dai, Ziye Wu, Mingsen Deng

**Affiliations:** School of Information, Guizhou University of Finance and Economics, Guiyang 550025, China

**Keywords:** molecular switches, bridging manner, dimethyldihydropyrene/cyclophanediene, density functional theory

## Abstract

A molecule–electrode interface with different coupling strengths is one of the greatest challenges in fabricating reliable molecular switches. In this paper, the effects of bridging manner on the transport behaviors of a dimethyldihydropyrene/cyclophanediene (DHP/CPD) molecule connected to two graphene nanoribbon (GNR) electrodes have been investigated by using the non-equilibrium Green’s function combined with density functional theory. The results show that both current values and ON/OFF ratios can be modulated to more than three orders of magnitude by changing bridging manner. Bias-dependent transmission spectra and molecule-projected self-consistent Hamiltonians are used to illustrate the conductance and switching feature. Furthermore, we demonstrate that the bridging manner modulates the electron transport by changing the energy level alignment between the molecule and the GNR electrodes. This work highlights the ability to achieve distinct conductance and switching performance in single-molecular junctions by varying bridging manners between DHP/CPD molecules and GNR electrodes, thus offering practical insights for designing molecular switches.

## 1. Introduction

The insatiable demand for smaller and faster devices has driven the rapid development of semiconductor technology in recent decades, leading to the emergence of new manufacturing techniques and functional component requirements. These novel components must be compatible with existing infrastructure to minimize costs. Therefore, one potential solution for achieving miniaturization in electronic devices is the utilization of molecular devices, which employ molecules as fundamental functional elements [1,2,3,4]. Due to their greater control flexibility compared to Si crystals, molecular systems offer opportunities for tailored functionality based on their unique chemical properties. This opens up possibilities for a wide range of molecular-based optoelectronic applications such as molecular switches [5,6,7], molecular rectifiers [8,9,10], negative differential resistance (NDR) devices [11,12,13] and spin filters [14,15,16].

A switch is an indispensable component for nearly all electronic devices. Molecular switches play a crucial role in fundamental information systems, such as logic gates and information storage [17,18]. Designing stable and reliable molecular switches has been a paramount challenge in the field of molecular electronics over the past two decades [19,20,21]. However, only a few molecular systems have demonstrated the ability to act as stable bidirectional switches during the process of electron conduction.

**Figure 1:**
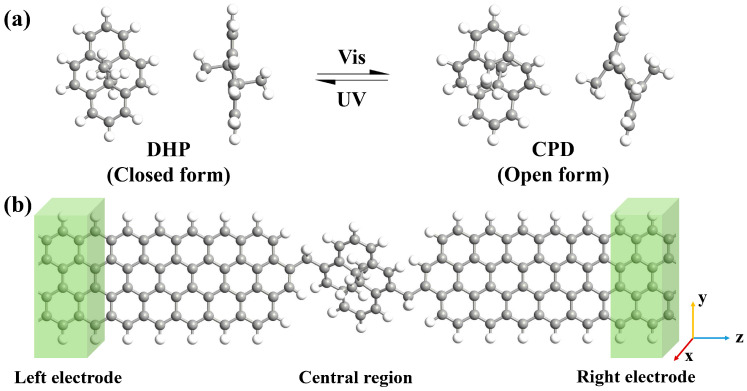
(**a**) Molecular geometries of DHP/CPD in front and side view. (**b**) Schematic diagram of DHP/CPD molecular junctions with GNR electrodes.

Photoresponsive molecules can be reversibly switched between two conductive states through light stimulation and are commonly utilized as functional units of molecular switches. Dimethyldihydropyrene/cyclophanediene (DHP/CPD) has been identified as a promising candidate for photoswitches [22,23,24], with the ring-closed form DHP and the ring-open form CPD being able to reversibly convert to each other via conrotatory electrocyclization under ultraviolet/visible (UV/Vis) light irradiation (Figure 1a). In 2013, Roldan et al. investigated the switching performance of a DHP derivative molecule using a mechanically controlled break junction (MCBJ) technique and found that the switching ratio between the two isomers could exceed 10^4^ [25]. In recent years, Han et al. discovered through theoretical simulations that incorporating an [e]-fusion of aromatic systems on a conjugated macrocycle in DHP/CPD-based molecular devices can enhance their switching performance, with benzo-fused molecular devices achieving up to a 10^3^ maximum switching ratio [26]. These works demonstrate the potential of the DHP/CPD molecule in the construction of high-performance molecular switching devices.

The conductance of molecular devices is influenced by various variables in addition to the inherent characteristics of the molecule. One of the key challenges in fabricating reliable molecular switches lies in obtaining molecule–electrode interfaces with different coupling strengths using various methods, such as self-assembled monolayers (SAMs) or MCBJs [27]. Numerous theoretical studies have also confirmed the significant influence of the molecule–electrode interaction on electronic transport. Berdiyorov et al. investigated the effect of halogen terminal groups on the electronic transport of aromatic and alkanethiolate molecules [28]. Zhang et al. discovered that linkage sites between molecules and gold electrodes dramatically modulate the switching performance of DHP/CPD junctions [29]. However, exposure to UV/Vis light can induce changes in the interactions between the core molecules and metallic electrode materials, which further impact the performance of molecular switches [30]. For example, the switching process in a diarylethene molecule junction self-assembled on gold electrodes is unidirectional and it can only change from the ring-closed form to the ring-open form, which can be attributed to the strong coupling between the diarylethene molecule and the gold electrodes [31]. Carbon-based materials such as carbon nanotubes and graphene have been alternatively employed in molecular devices for achieving stable and controlled contact [32,33,34,35]. Graphene nanoribbons (GNRs) possess high electron mobility and stable chemical properties, making them ideal replacements for traditional electrode materials. However, connecting molecules with GNR electrodes involves various bridging configurations, resulting in a more complex electronic structure and molecule–electrode interaction compared to molecule–metal electrode interfaces. Jia et al. successfully achieved a stable bidirectional reversible single-molecule switch by introducing three CH_2_ groups between the diarylethene molecule and GNR electrodes to weaken the molecule–electrode coupling [36]. Yang et al. reported, from theoretical simulations, that different lateral linking groups including –BO_2_, –CH_3_, –COOH, –NH_2_ and –OH significantly affect conductance and switching ratio in biphenyl-2,2′-dithiol devices with GNR electrodes [37]. Therefore, understanding the effect of the molecule–GNR electrode interaction on the performance of molecular switches is of utmost importance in order to design and fabricate highly stable and high-performance molecular switches.

In this work, inspired by the aforementioned experimental and theoretical works, we aim to investigate the effect of the bridging manner between the molecule and the GNR electrodes on the electronic transport and switching performance of photoswitches. The DHP/CPD photochromic isomers are chosen as our test bed with GNRs serving as electrodes. The linkage groups consist solely of carbon and hydrogen atoms, including CH_2_, CH=CH and C≡C groups. Additionally, we also consider the influence of the electrode edge structure. The electric transport and switching properties of single-molecular junctions, where the DHP/CPD molecule is positioned between two GNR electrodes in different bridging manners, are investigated by using the nonequilibrium Green’s function (NEGF) method in combination with density functional theory (DFT). Our computational results demonstrate that altering the bridging manner significantly modulates the electronic transport behaviors of DHP/CPD molecular junctions, including conductance and current switching ratio. These modulations in transport properties can be attributed to variations in energy alignment between the electronic states of the molecule and the electrodes caused by bridging manner.

## 2. Results and Discussion

**Figure 2:**
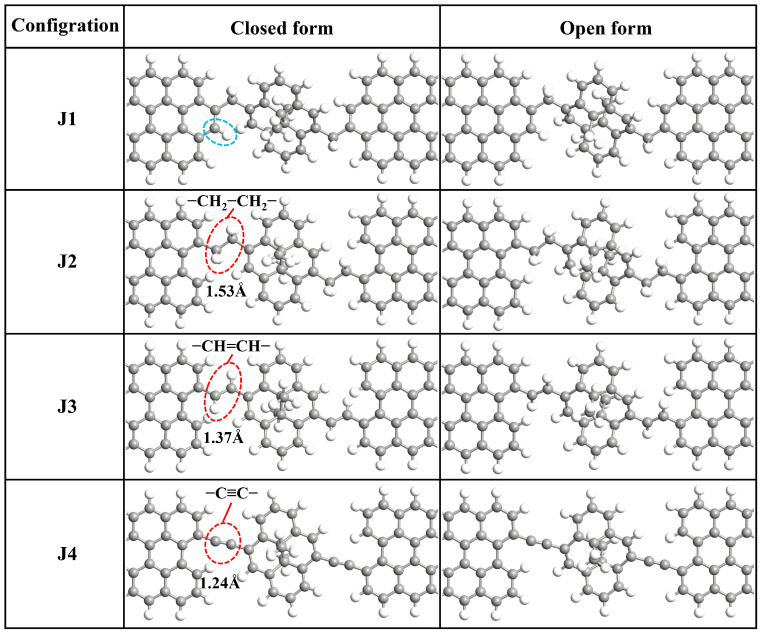
Geometric structures of the molecular junctions J1~J4. The gray and white balls represent C and H atoms, respectively. J1 and J2 are constructed by connecting a DHP/CPD to GNRs by CH_2_ groups, except there is a vacancy at an edge C atom (circled by the blue dashed line) at the electrode in J2 and the C atoms connected to it are all saturated by H atoms. J3 and J4 have the same electrode edge structure as J2, except the groups between molecule and electrodes are different (circled by the red dashed lines).

The schematic diagram of the molecular junction in this work is shown in Figure 1b. The molecular junction can be divided into three parts: the left electrode, the central region and the right electrode. Obviously, there are four bridging configurations of DHP/CPD contacted to zigzag graphene nanoribbon (ZGNR) electrodes as shown in Figure 2. The bridging manners are named as J1, J2, J3 and J4 configuration respectively, where J3 is the bridging manner adopted by Han et al. in similar DHP/CPD molecular junctions [26]. The closed/open forms of J1 to J4 configurations of the molecular junctions are denoted as J1c/J1o to J4c/J4o for convenience. It is evident that J1 and J2 exhibit distinct electrode edge structures, wherein a vacancy at the C atom at the electrode in J2 exists, with all connected C atoms being saturated by H atoms. Meanwhile, J2, J3 and J4 share identical electrode edge structures, except for the bonding between the molecule and the GNR electrodes via C-C, C=C and C≡C bonds, and the optimized bond lengths are 1.53 Å, 1.37 Å and 1.24 Å respectively. These values directly reflect variations in bridging strength between the molecule and the electrodes. Consequently, we can investigate how the electrode edge structure and covalent bond strength between molecule and electrode influence the transport behavior through the four different bridging manners.

**Figure 3:**
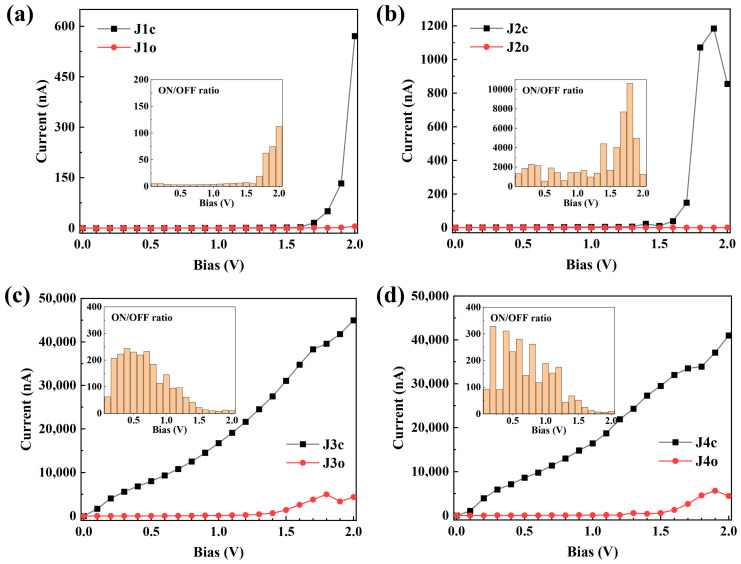
Calculated current–voltage (I–V) characteristics and ON/OFF ratios of the molecular junction J1 (**a**), J2 (**b**), J3 (**c**) and J4 (**d**).

The calculated current–voltage (I–V) characteristics of the molecular junctions in the bias voltage range of [0, 2.0 V] are presented in Figure 3. It can be observed that the current values of J1c and J2c gradually increase between 0 V and 1.5 V before experiencing a rapid increase beyond 1.5 V (Figure 3a,b). J1o and J2o exhibit negligible current variations throughout the entire bias range. The current of J3c demonstrates a noticeable increase across the entire bias range, while the current of J3o exhibits a slower trend compared to J3c and only shows significant enhancement when the bias exceeds 1.3 V (Figure 3c). Similar variations in current values are observed for J4c and J4o as seen for J3. Notably, both J1 and J2, where molecules are connected to electrodes via CH_2_ groups, exhibit, as expected, significantly lower currents than J3 and J4, which are connected via conjugated groups. For instance, at a bias voltage of 2.0 V, the current values for ring-closed configurations (J1c~J4c) are 571 nA, 855 nA, 44,960 nA and 41,000 nA, respectively. With only a difference in bridging strength, the tunneling current in J3c is more than 52 times larger than that in J2c at a bias voltage of 2.0 V alone. Moreover, the current magnitude flow through J3c is nearly 80 times greater than that through J1c. For the ring-open configuration, the current values of J1o~J4o are 5 nA, 0.7 nA, 4366 nA and 4350 nA, respectively. The bridging manner exhibits a current modulation exceeding three orders of magnitude, particularly with J3o and J4o displaying even higher currents than J1c and J2c. This result suggests that the electronic conductance of DHP/CPD molecular junctions is significantly influenced by the manner in which they are bridged.

The current through the ring-closed configurations is consistently observed to be larger than that of the ring-open forms, thereby indicating significant switching effects in J1~J4 junctions (Figure 3). To quantitatively characterize the switching performance of these devices, we define the current ON/OFF ratio as follows: $R(V)=I_{close}/I_{open}$, where *I*_close_ and *I*_open_ represent the current values through the ring-closed and ring-open forms at the same bias *V*, respectively. As shown in Figure 3, J1~J4 exhibit maximum ON/OFF ratios of 112, 10,623, 244 and 328, respectively. Notably, J2 achieves an exceptionally high ON/OFF ratio exceeding 500 across the entire range of biases (Figure 3b), whereas J1 only achieves a maximum value of 112 with a difference only in electrode edge. In contrast, J3 and J4 demonstrate better switching performance (ON/OFF ratio > 100) within the bias range [0.1, 1.0 V] and [0.1, 1.2 V], respectively, compared to the higher bias range due to the pronounced increase in current at high bias voltages for J3o and J4o (Figure 3c,d). These results indicate that the switching performance of DHP/CPD devices can be significantly modulated by altering the bridging manner. In addition, the zero-bias transmission at Fermi level (*E*_F_) of J1~J4 is shown in Table 1, where the zero-bias transmission ratio is defined as: $T_{close}/T_{open}$. According to Table 1, the order of $T_{close}/T_{open}$ is ranked as: J2 > J4 > J3 > J1, perfectly aligning with their respective maximum ON/OFF ratios. This suggests that the zero-bias transmission ratio can also be used to assess switching performance effectively.

**Figure 4:**
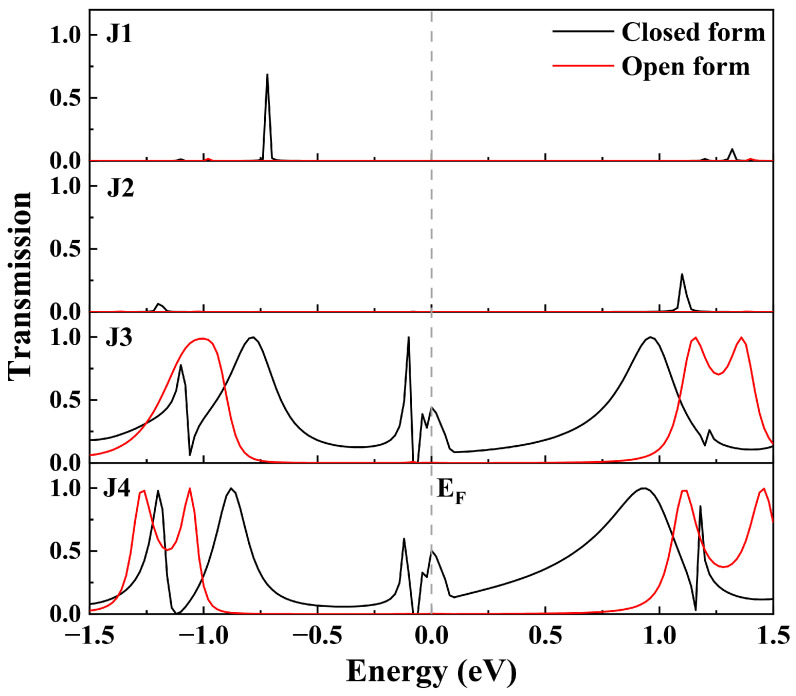
Transmission spectra of the closed form and open form for J1~J4 junctions at zero bias. The *E*_F_ is set at zero on the energy scale.

**Figure 5:**
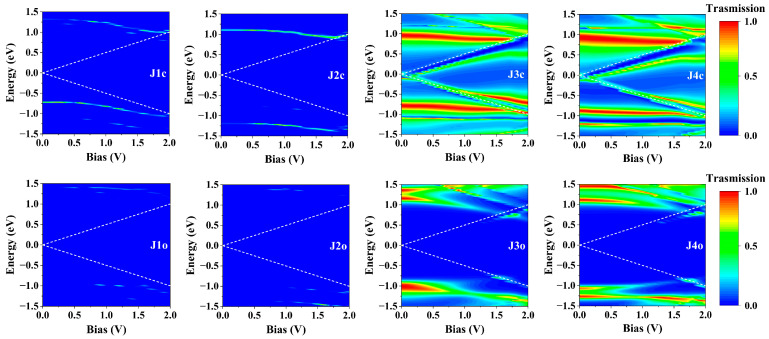
Bias-dependent transmission spectra of J1~J4 junctions. The white dashed lines in each subgraph represent the bias window.

To elucidate the origin of the conductance disparity among the molecular junctions, we have performed calculations on the zero-bias transmission spectra (Figure 4) and the bias-dependent transmission spectra (Figure 5) of J1~J4. As shown in Figure 4, both J1c and J2c exhibit two distinct narrow transmission peaks within the energy range [−1.5, 1.5 eV], which are significantly distanced from the *E*_F_, indicating their limited conductance at low bias voltage. With the increase of bias voltage, the transmission peaks around 1.2 eV of J1c and J2c gradually shift towards and eventually enter into the bias window after reaching 1.5 V, resulting in a rapid increase in current flow (as shown in Figure 3a,b). Conversely, no discernible transmission peak is observed within the energy range [−1.5, 1.5 eV] at zero bias for either J1o or J2o (Figure 4), even when the bias increases to 2.0 V, hence explaining their low conductance within the [0, 2.0 V] bias range (Figure 3a,b). In contrast, multiple broad and strong transmission peaks can be identified in the spectra of J3c and J4c and, in particular, the peaks at *E*_F_ play a major role in low-bias electronic transport (Figure 4). With an increasing bias voltage, the transmission peak at *E*_F_ for J3c and J4c splits into two distinct peaks, which remain within the bias window while exhibiting enhanced intensity and width; this is the driving force behind the continuous increase in current flow through J3c and J4c (Figure 5). It is noteworthy that, despite the utilization of diverse simulation details, the transmission spectral characteristics of J3 exhibit consistency with the computational findings reported by Han et al. for similar molecular junctions [26], thereby substantiating the reliability of our calculations. In contrast, the transmission spectra of J3o and J4o exhibit no transmission peaks at low biases within the bias window (Figure 5). However, after reaching 1.5 V, two transmission peaks at about -0.8 eV and 0.7 eV enter the bias window, thereby enhancing the current under high bias conditions and reducing the ON/OFF ratio in J3 and J4 (Figure 3c,d). Consequently, it is evident that the transmission spectra of J1 and J2 differ significantly from those of J3 and J4, indicating that both the broadening and intensity of the transmission peaks depend on the bridging manner. In addition, despite having different bonding strengths, similar conductive properties are observed between J3 and J4 due to their comparable transmission spectra.

**Figure 6:**
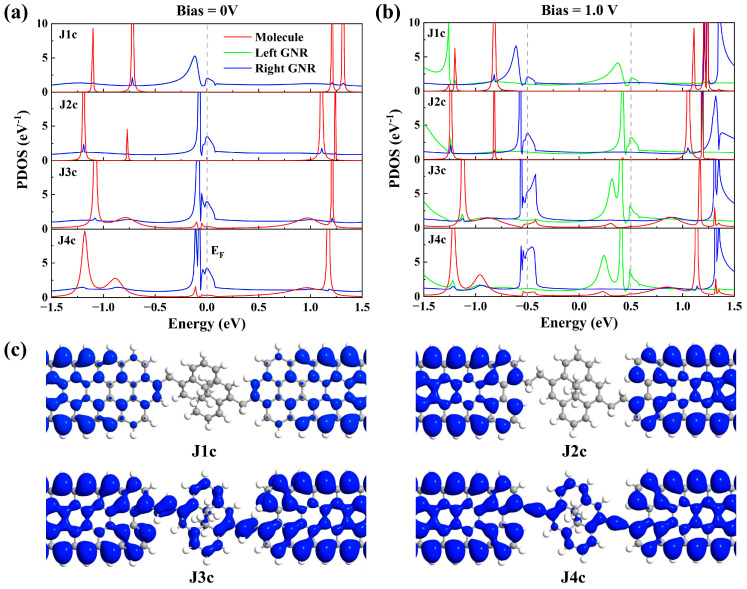
Projected density of states (PDOS) spectra for the central region of J1c~J4c junctions when the bias voltage is set to 0 V (**a**) and 1.0 V (**b**). The projection subspace of the scattering region can be divided into three parts, i.e., the left GNR (including the left bridging group), the molecule and the right GNR (including the right bridging group). Local density of states (LDOS) for the central region at *E*_F_ under 0 V bias (**c**). An isovalue of 0.005 is chosen for all plots.

From the comparison of transmission spectra, it is found that the difference in conductance between these molecular junctions with different bridging manners mainly depends on the transmission peak near *E*_F_. To further reveal the origin of these transmission peaks, the projected density of states (PDOS) spectra of J1c~J4c under 0 V and 1.0 V bias are plotted to demonstrate the contribution of each component to the transmission peaks (Figure 6a,b). As depicted in Figure 6a, strong PDOS peaks at *E*_F_ originating from the GNR electrodes are observed in all four junctions, while the PDOS peaks at *E*_F_ contributed by the molecule are only detected in J3c and J4c, albeit with weak intensity. Notably, distinct differences can be observed in the shape of PDOS peaks contributed by the GNR electrodes between J1c and other junctions due to variations in electrode edge structure. With an increase in bias voltage, all PDOS peaks at *E*_F_ split into two separate peak groups and shift towards the boundaries of the bias window (Figure 6b), which aligns with characteristics exhibited in the transmission spectra of J3c and J4c. Meanwhile, the PDOS peaks contributed by the GNR electrodes (especially the left GNR) in J3c and J4c exhibit multiple peaks and a broader energy distribution, thereby overlapping with the split peaks of the molecule (at about -0.4 eV and 0.25 eV). However, no PDOS peaks contributed by the molecule are observed within the bias window for J1c and J2c. This indicates that the transmission peak is strongly dependent on the overlapping region of the PDOS peaks originating from the molecule and electrodes that reflects the energy-matching degree of electronic state between them. The local density of states (LDOS) distributions provide further clarity on this matter, as depicted in Figure 6c, as the zero-bias electronic states at *E*_F_ of J1c and J2c are all localized on the electrodes, thus failing to establish an electron transmission channel. In contrast, the electronic states of J3c and J4c are distributed across both electrodes and the molecule; such delocalized electronic states are favorable for electron transport. Therefore, the electronic state distribution on the molecule plays a pivotal role in facilitating electron transmission. The establishment of an effective channel for electron transport necessitates matching energy levels between the molecule and electrodes, and altering the bridging manner can modulate the energy level alignment between the molecule and electrodes. This elucidates why introducing CH_2_ groups weakens the molecule–electrode coupling [36,38,39], as it reduces the energy level matching between the molecule and electrodes.

Through the density of state analysis, it has been observed that the electronic state distribution of the same molecule undergoes change due to the influence of the bridging manner. To clarify the influence of the bridging manner on the molecular electronic structures, the molecule-projected self-consistent Hamiltonians (MPSH) technique is utilized to compute the eigenvalues of DHP/CPD in J1~J4 (presented in Table 2). In this analysis, the self-consistent Hamiltonian of the entire junction is projected onto the molecule moiety, excluding the electrodes and bridging groups, to assess the influence of different bridging manners on the eigenvalues of identical molecules. As shown in Table 2, the highest occupied molecular orbital (HOMO) and the lowest molecular orbital (LUMO) of the same molecule are shifted due to different bridging manners. Specifically, the LUMO energies of J3 and J4 are lower than those of J1 and J2, resulting in smaller energy gaps. The energy gaps for closed/open forms with distinct bridging manners follow this order: J1 > J2 > J4 > J3, which is generally consistent with the current magnitude trend (Figure 3). For the same bridging manner, the energy gap of the ring-open configuration is always larger than that of the closed one due to the destruction of the central benzene rings during DHP conversion to CPD, resulting in reduced planar conjugation within the molecule (Figure 1a). Therefore, the relationship between the HOMO–LUMO gap and conductance in molecular junctions J1~J4 can be simply summarized as follows: a smaller energy gap corresponds to higher conductance. This observation is consistent with our previous findings, as molecules with smaller energy gaps exhibit energy levels that are in closer proximity to the *E*_F_ of electrodes, which means that the energy levels between the molecule and the electrodes are better matched.

**Figure 7:**
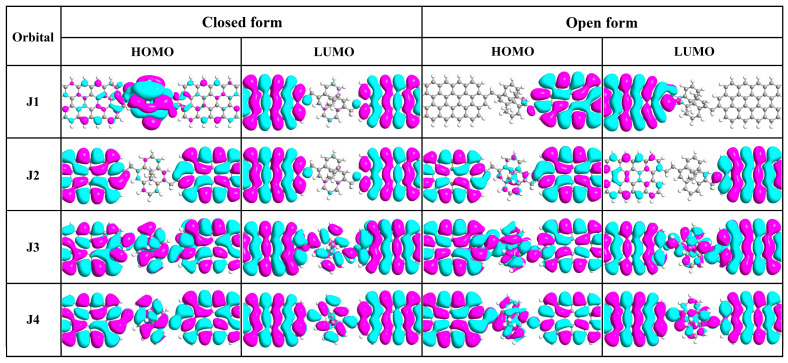
Spatial distribution of the frontier molecular orbitals on the central regions of J1~J4 junctions at zero bias. The isovalue is set to 0.01 for all plots.

Furthermore, the self-consistent Hamiltonian of J1~J4 junctions are projected onto the central regions to calculate the spatial distribution of frontier molecular orbitals to display the interaction between the GNR electrodes and the DHP/CPD molecule more clearly (as shown in Figure 7). It is clear that the HOMO and LUMO of J1 and J2 junctions, including the closed and open form, are predominantly localized at the electrodes or molecule segment, which serve as nonconducting channels. Conversely, both the HOMO and LUMO of J3 and J4 exhibit delocalization across the entire junction, enabling more efficient electron transport. It is worth noting that the orbitals localized on the molecule (the HOMO of J1c) are delocalized on the molecular moiety, and those localized on the electrode (the other orbitals of J1 and J2) are also delocalized on their respective electrode parts, thus indicating that their localization characteristics primarily depend on the bridging group. The spatial distribution of frontier molecular orbitals closely resembles that of the LDOS distribution of J1c~J4c (Figure 6c), further demonstrating that the bridging CH_2_ group can weaken the molecule-electrode interaction, while conjugated CH=CH and C≡C groups facilitate stronger molecule-electrode coupling.

## 3. Computational Methods

All calculations are performed by DFT and NEGF methods implemented in QuantumATK software (version P-2019.03-SP1) [40]. The Perdew–Burke–Ernzerhof (PBE) functional [41] of the generalized gradient approximation (GGA) is used to calculate the exchange and correlation energies of valence electrons, while the optimized norm-conserving Vanderbilt pseudopotentials [42] are adopted to describe the ionic nuclei. The mesh cutoff is set to 250 Ry and a 1 × 1 × 300 grid is chosen for the Brillouin-zone *k*-sampling. To prevent the influence of boundary conditions in the *x*- and *y*-axes perpendicular to the electron transmission direction, we take a vacuum layer of 15 Å in these two directions.

The tunneling current through the molecular junctions can be calculated by the Landauer–Büttiker formula [43]:

(1) $$I\left(V\right)=\frac{2e}{h}\int T\left(E,V\right)\left[f\left(E-\mu_{L}\right)-f\left(E-\mu_{R}\right)\right]dE$$

where *V* is the bias voltage applied to the molecular junctions, *T*(*E*, *V*) is the bias-dependent transmission coefficient, $f\left(E-\mu_{L}\right)$/$f\left(E-\mu_{R}\right)$ is the Fermi–Dirac distribution function of the left/right electrodes and $\mu_{L}$/$\mu_{R}$ is the chemical potential of the left/right electrode.

## 4. Conclusions

In summary, we have investigated the electronic transport properties of four all-carbon molecular junctions, wherein a DHP/CPD molecule is connected to GNR electrodes in different bridging manners. Our calculations demonstrate that the electronic transport and switching performance of the molecular junctions are significantly influenced by the bridging manner. By altering the structure of the electrode edge and the bond level of the bridging group, both current values and ON/OFF ratios can be modulated to more than three orders of magnitude. Particularly for the molecular junction connected by a C–C bond with an edge C atom vacancy at the electrode, an ON/OFF ratio exceeding 10^3^ is achieved over a wide bias voltage range. The junctions connected by C=C bonds and C≡C bonds exhibit similar conductance, which significantly surpass that of C–C bond connections, and display better switching performance (ON/OFF ratio > 100) in the low bias range (<1.0 V). Importantly, altering the bridge manner can modulate the energy level alignment between the molecule and the electrodes, which is the fundamental determinant for the change in conductance of a single-molecule junction. This study demonstrate how varying bridging configurations between electrodes and core molecules can yield distinct transport and switching characteristics in single-molecule junctions, thereby highlighting the importance of the choice of bridging manner when designing molecular devices.

## Figures and Tables

**Table 1:** Zero-bias transmission at *E*_F_ of J1~J4 junctions.

	J1	J2	J3	J4
*T*_close_	4.44 × 10^−6^	1.46 × 10^−4^	0.44	0.50
*T*_open_	2.66 × 10^−6^	2.41 × 10^−7^	2.17 × 10^−3^	2.28 × 10^−3^
*T*_close_/*T*_open_	2	606	203	219

**Table 2:** MPSH eigenvalues of DHP/CPD molecule moiety in J1~J4 junctions.

MPSH Eigenvalues (eV)	Closed Form			Open Form		
	HOMO	LUMO	Gap	HOMO	LUMO	Gap
J1	−0.74	1.17	1.91	−1.02	1.38	2.40
J2	−0.76	1.10	1.86	−0.36	1.67	2.03
J3	−0.72	0.56	1.28	−1.02	0.72	1.74
J4	−0.66	0.74	1.40	−1.12	0.84	1.96

## Data Availability

Data are available upon request.
